# Commissural and monosynaptic inputs to medial vestibular nucleus GABAergic neurons in mice

**DOI:** 10.3389/fneur.2024.1484488

**Published:** 2024-10-08

**Authors:** Dedi Kong, Lingxi Kong, Chengwei Liu, Qianru Wu, Jing Wang, Chunfu Dai

**Affiliations:** ^1^Department of Otology and Skull Base Surgery, Eye Ear Nose and Throat Hospital, Fudan University, Shanghai, China; ^2^Key Laboratory of Hearing Medicine, Ministry of Health, Eye Ear Nose and Throat Hospital, Fudan University, Shanghai, China; ^3^Department of Pharmacology, School of Basic Medical Sciences, State Key Laboratory of Medical Neurobiology and MOE Frontiers Center for Brain Science, and Institutes of Brain Science, Fudan University, Shanghai, China

**Keywords:** vestibular function, vestibular disorders, vestibular compensation, medial vestibular nucleus, GABAergic neurons

## Abstract

**Objective:**

MVN GABAergic neurons is involved in the rebalance of commissural system contributing to alleviating acute peripheral vestibular dysfunction syndrome. This study aims to depict monosynaptic inputs to MVN GABAergic neurons.

**Methods:**

The modified rabies virus-based retrogradation method combined with the VGAT-IRES-Cre mice was used in this study. Moreover, the commissural connections with MVN GABAergic neurons were analyzed.

**Results:**

We identified 60 nuclei projecting to MVN GABAergic neurons primarily distributed in the cerebellum and the medulla. The uvula-nodulus, gigantocellular reticular nucleus, prepositus nucleus, intermediate reticular nucleus, and three other nuclei sent dense inputs to MVN GABAergic neurons. The medial (fastigial) cerebellar nucleus, dorsal paragigantocellular nucleus, lateral paragigantocellular nucleus and 10 other nuclei sent moderate inputs to MVN GABAergic neurons. Sparse inputs to MVN GABAergic neurons originated from the nucleus of the solitary tract, lateral reticular nucleus, pedunculopontine tegmental nucleus and 37 other nuclei. The MVN GABAergic neurons were regulated by the contralateral MVN, lateral vestibular nucleus, superior vestibular nucleus, and inferior vestibular nucleus.

**Conclusion:**

Our study contributes to further understanding of the vestibular dysfunction in terms of neural circuits and search for new strategies to facilitate vestibular compensation.

## 1 Introduction

The medial vestibular nuclei (MVN), is a crucial processor of vestibular inputs (1). These inputs primarily originate from crista ampullaries of two lateral semicircular canals (2). The MVN integrates information regarding the head movement in space. In addition, visual, and proprioceptive signals also converge in the MVN (3, 4). The MVN sends ascending axonal fibers to the oculomotor nuclei mediating the vestibuloocular reflex (VOR) and bilateral descending projections to the cervical ventral horn to control the vestibular-spinal reflex. Thus, it is essential in maintaining posture, clear vision and static and dynamic balance (5, 6). Furthermore, it is also involved in cognition, such as navigation, spatial memory and learning (7). Normal vestibular function is essential for daily life. When patients suffer from vestibular dysfunction, they complain acute vestibular syndrome (8). It is characterized by vertigo, gaze instability, vegetative disorders, and cognitive alterations which strongly limit daily activities (9, 10). Certain syndrome can alleviate over time is known as vestibular compensation (11). However, the mechanisms underlying vestibular compensation remain unclear.

In the rhombomeric perspective, the MVN in mouse extends at least from rhombomere r5 to r6. The MVN is comprised of two heterogeneous divisions: small dorsal neurons and larger ventral neurons. Cells in both divisions of the MVN express GAD67 mRNAs which labels cell bodies of GABAergic neurons (12, 13). Previous immunohistochemical studies demonstrated that dorsal neurons in the MVN synthesize gamma-aminobutyric acid (GABA) and are intensely stained by GABA-antibody, supporting a functional GABAergic system exists within the MVN (14–16). GABA is considered as a common inhibitory neurotransmitter within brain (17). Further studies had revealed that GABAergic neurons produced regular firing in electrophysiological recording technology (18–20).

GABAergic neurons within the MVN send axons to the cervical ventral horn and the oculomotor nuclei to mediate the inhibitory influence (21, 22). MVN GABAergic neurons project primarily to the caudal ventrolateral medulla (CVLM) to mediate vestibulosympathetic reflex, showing a target preference (23). Moreover, MVN GABAergic neurons are essential for vestibular compensation by involving in the commissural system between the bilateral MVN (17, 24–26).

Taken together, GABAergic neurons within the MVN are involved in controlling posture, balance, and gaze stabilization, particularly in vestibular compensation. Thus, investigating the afferent inputs to MVN GABAergic neurons will facilitate the search for optional circuits that manipulate GABAergic neurons. The connectivity of MVN neurons have been previously investigated using classic retrograde and anterograde tracers. These studies showed that projections to MVN originated from the dorsal raphe nucleus, inferior olivary, and parabrachial nucleus (27–31). However, specific inputs to MVN GABAergic neurons remain unelucidated. Unlike traditional tracers that cannot distinguish neuron types, the current modified rabies virus (RV) method and the Cre/LoxP system enable to identify specific neurons without affecting passing neural tracts. Accordingly, it allows us to explore neural connectivity of a well-defined neuron type rather than a specific brain region (32–35). In this study, we used modified RV and VGAT-IRES-Cre mice to map out monosynaptic inputs targeting MVN GABAergic neurons.

## 2 Materials and methods

### 2.1 Animals

Adult VGAT-IRES-Cre mice and their wild-type littermates were used in this study. All mice were housed under suitable environment (constant temperature: 22 ± 0.5°C and relative humidity: 60% ± 2%) and ensured an adequate supply of food and water. All animal experiments were approved by the Animal Experiments Ethics Committee at Shanghai Public Health Clinical Center, Fudan University.

### 2.2 Virus

All viruses used in the retrograde tracing study were acquired from BrainVTA (Wuhan, China). rAAV2/9-Ef1α-DIO-EGFP-TVA-WPRE (5 × 10^12^ genomic copies/mL) and rAAV2/9-Ef1α-DIO-RVG-WPRE (5 × 10^12^ genomic copies/mL) were combined in equal proportions as the helper virus. And the titer of the RV-ENVA-ΔRG-DsRed (RV) was 2 × 10^8^ genomic copies/mL.

### 2.3 Virus injection and histological preparation

Virus injection and histological preparation were performed as previously described (32, 33). All mice undergone twice injections of virus injections, respectively. Brief description as following, anesthetized VGAT-IRES-Cre and wild-type mice (pentobarbital sodium, 50 mg/kg, intraperitoneal) were securely positioned on a stereotaxic instrument (RWD Life Science, China). And its skull was aligned to make it parallel to the reference plane. Firstly, 100 nL of the AAV-helper virus mixture were injected into the unilateral MVN (−6.0 mm AP, +0.8 mm ML, −3.2 mm DV). Three weeks afterward, double volume of RV was injected into the same position as before. An additional 10 min of holding the pipette was required to ensure full diffusion of virus particles into the target nuclei.

One week later, the anesthetized mice were perfused with 0.1 M phosphate-buffered saline, then with 4% paraformaldehyde. The brain samples were post-fixed in 4% paraformaldehyde overnight. Subsequently, they were dehydrated in various gradients (10, 20, 30%) of sucrose. Brain samples were coronally sectioned (30-μm thick). All samples were divided into three series.

### 2.4 Imaging and data analysis

All sections were imaged by virtual-slide microscope (Olympus, Tokyo, Japan). The Olympus analysis software (OlyVIA v.2.9, Tokyo, Japan) and ImageJ software (v.2.1.0, Bethesda, MD, United States) were utilized for detailed analyses. Starter cells were identified by co-expressing DsRed and GFP, whereas afferent neurons only expressed DsRed. Brain structures were recognized based on the standard atlas of mouse brain (36). The neurons labeled with DsRed were counted. To quantify ipsilateral afferent inputs, the input from each nucleus was quantified relative to the total number of input neurons. All data are presented in the form of mean ± standard error of the mean (SEM).

## 3 Results

### 3.1 Approaches for identifying monosynaptic inputs to MVN GABAergic neurons

The modified RV-based tracing system was utilized with VGAT-IRES-Cre mice in this study. The helper viruses were Cre-dependent, they can only infect the GABAergic neurons with Cre recombinase. Thus, the enhanced green fluorescent protein (EGFP), avian-specific retroviral receptor (TVA), and the rabies glycoprotein G (RG) were specifically expressed on GABAergic neurons. The modified RV with an avian virus envelope protein (EnvA) only infects neurons with TVA and spread retrogradely with the help of RG. Accordingly, the genetically modified RV retrograde tracing system combined with VGAT-IRES-Cre mice were used to map the afferent inputs to MVN GABAergic neurons (32–35).

**Figure 1:**
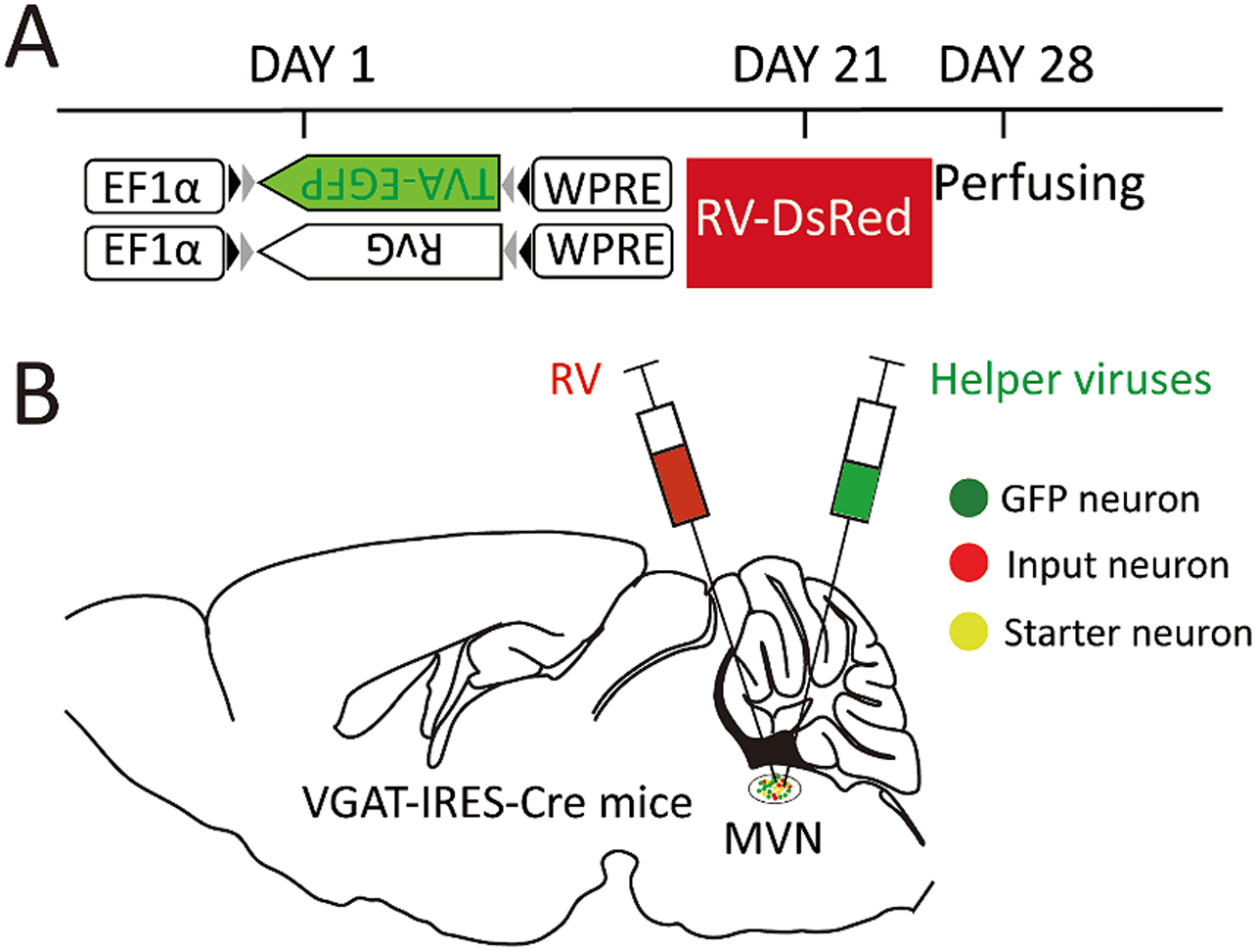
Experimental strategy for RV-based retrograde tracing in MVN GABAergic neurons. **(A)** A schematic diagram illustrating the viral vectors and injection steps for virus; **(B)** A schematic diagram showing the injection site into the MVN of VGAT-IRES-Cre mice.

On the first day, the helper virus (100 nL) was administered into the unilateral MVN of the wide-type and VGAT-IRES-Cre mice. These Cre-dependent viruses can exclusively infect GABA neurons where the Cre recombinase was present. Then GABA neurons infected with helper virus express TVA-GFP and RG proteins. After 3 weeks, double volume of RV was administered into the previous location. One week later, all mice were sacrificed and perfused (Figure 1).

**Figure 2:**
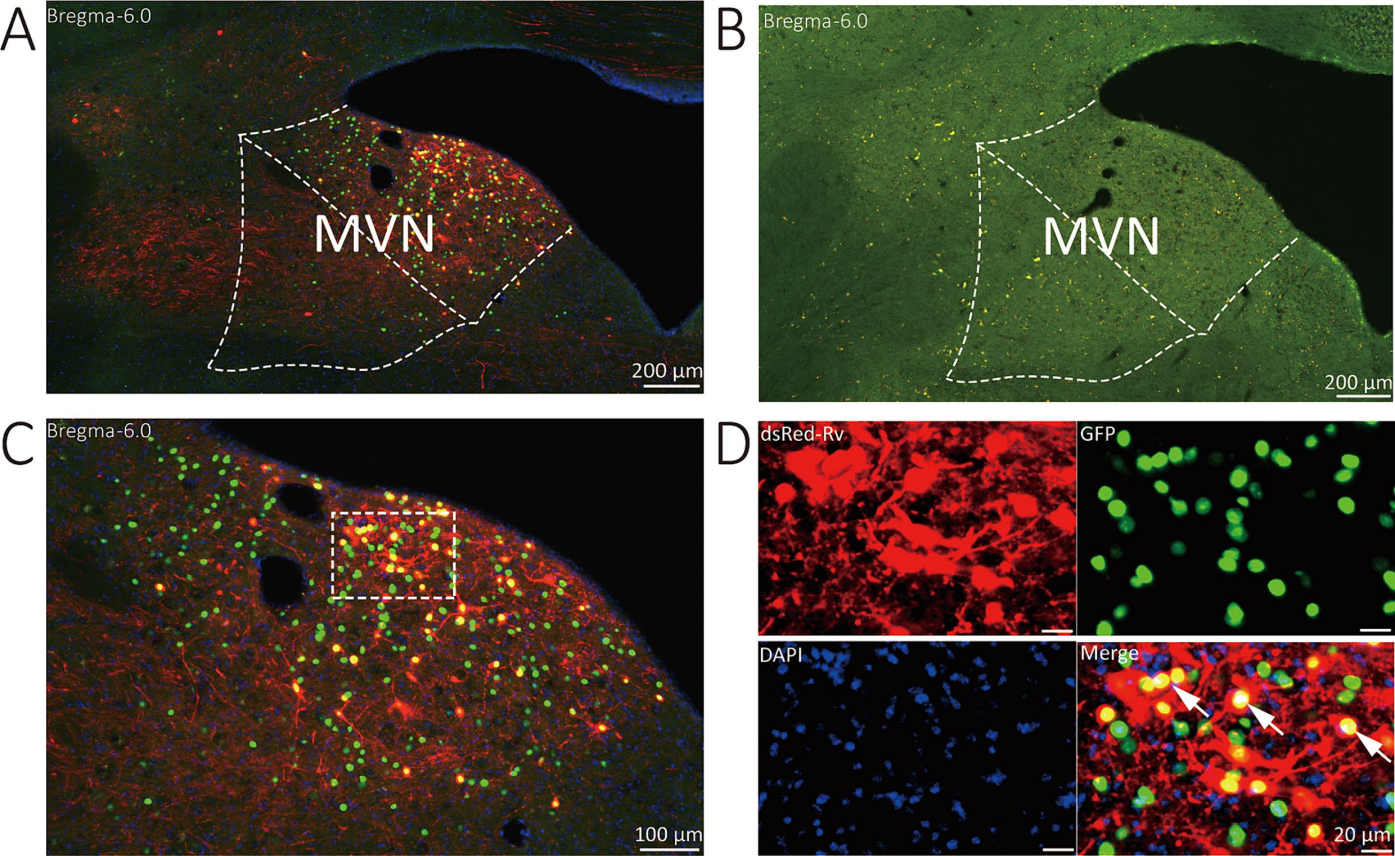
Representative images of MVN GABAergic neurons injected with tracing virus. **(A)** Injection site of the unilateral MVN of VGAT-IRES-Cre mice. **(B)** Injection site of the unilateral MVN of wide-type mice. **(C,D)** Representative images displaying starter neurons (yellow), helper viruses-labeled neurons (green) and input neurons (red). The white arrows show the starter neurons.

The starter neurons were described as expressing both GFP and DsRed. Three types of neurons (GFP-labeled neurons, DsRed-labeled neurons, and start neurons labeled by both GAP and DsRed) were observed in the MVN of VGAT-Cre mice. Wild-type littermates without the Cre recombinase were used to verify virus specificity. In the MVN of wild-type mouse, neither GFP nor DsRed-positive cells were found (Figure 2).

### 3.2 Overview of monosynaptic inputs to MVN GABAergic neurons

**Figure 3:**
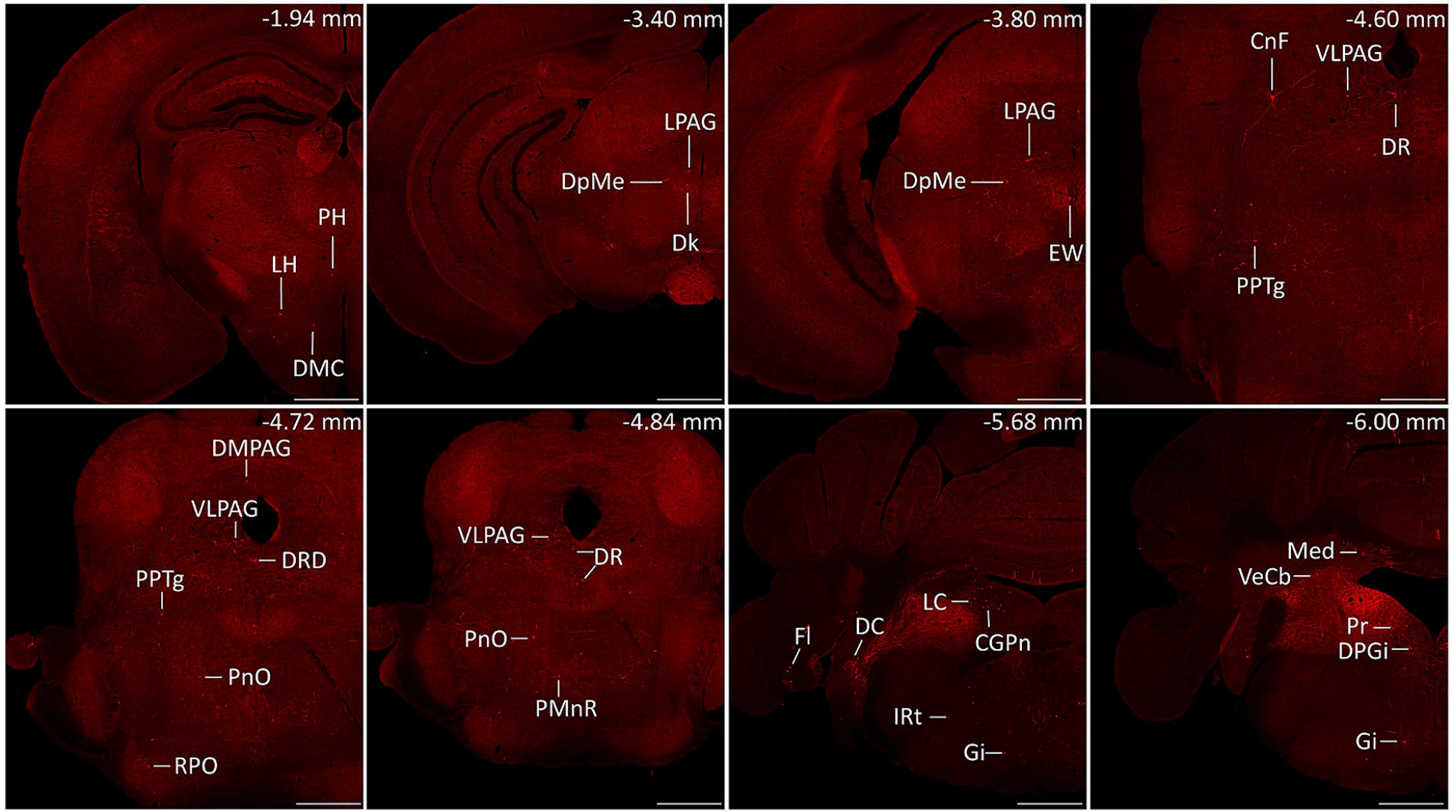
Representative images of monosynaptic inputs to MVN GABAergic neurons. Brain structures were determined according to the standard mouse atlas. Only the ipsilateral hemisphere was shown. Scale bar: 500 μm. CGPn, central gray of the pons; CnF, cuneiform nucleus; DC, dorsal cochlear nucleus; Dk, nucleus of Darkschewitsch; DMC dorsomedial hypothalamic nucleus, compact part; DMPAG, dorsomedial periaqueductal gray; DPGi, dorsal paragigantocellular nucleus; DpMe, deep mesencephalic nucleus; DR, dorsal raphe nucleus; DRD dorsal raphe nucleus, dorsal part; EW, Edinger-Westphal nucleus; Fl, flocculus; Gi, gigantocellular reticular nucleus; IRt, intermediate reticular nucleus; LC, locus coeruleus; LH, lateral hypothalamic area; LPAG, lateral periaqueductal gray; Med, medial (fastigial) cerebellar nucleus; PH, posterior hypothalamic area; PMnR, paramedian raphe nucleus; PnO, pontine reticular nucleus, oral part; PPTg, pedunculopontine tegmental nucleus; Pr, prepositus nucleus; RPO, rostral periolivary region; VeCb, vestibulocerebellar nucleus; VLPAG, ventrolateral periaqueductal gray.

**Figure 4:**
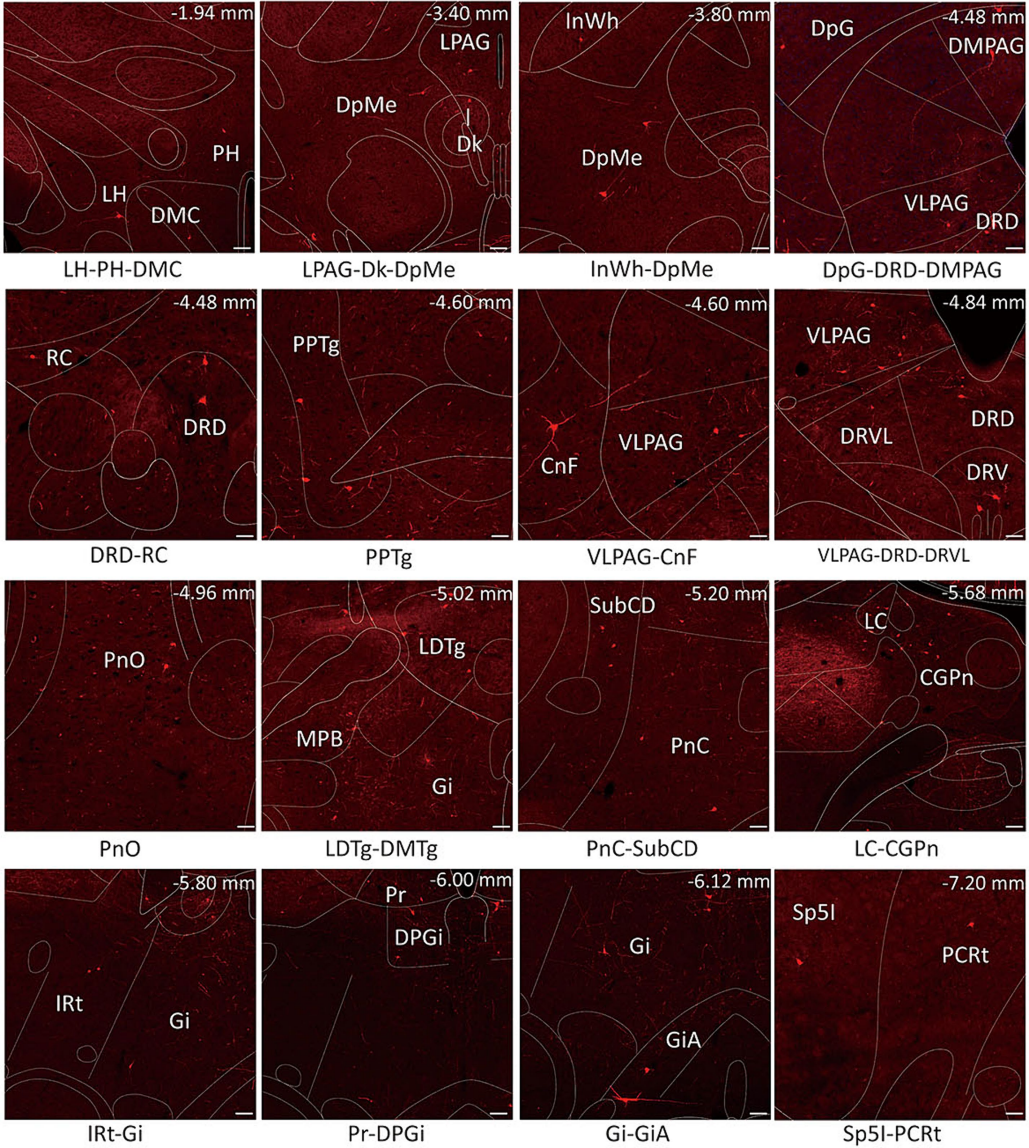
Schematic images of the functional regions with monosynaptic inputs to MVN GABAergic neurons. Primary inputs to MVN GABAergic neurons originated from brain regions associated with oculomotor controlling [e.g., flocculus, medial (fastigial) cerebellar nucleus and prepositus nucleus], sleep–wake regulation (e.g., dorsal paragigantocellular nucleus, lateral paragigantocellular nucleus and ventrolateral periaqueductal gray) and sympathetic response (e.g., gigantocellular reticular nucleus and intermediate reticular nucleus). Scale bar: 100 μm. CGPn, central gray of the pons; CnF, cuneiform nucleus; Dk, nucleus of Darkschewitsch; DMC,dorsomedial hypothalamic nucleus, compact part; DpG, deep gray layer of the superior colliculus; DPGi, dorsal paragigantocellular nucleus; DpMe, deep mesencephalic nucleus; DRD, dorsal raphe nucleus, dorsal part; DRV, dorsal raphe nucleus, ventral part; DRVL, dorsal raphe nucleus, ventrolateral part; Gi, gigantocellular reticular nucleus; GiA, gigantocellular reticular nucleus, alpha part; InWh, intermediate white layer of the superior colliculus; IRt, intermediate reticular nucleus; LC, locus coeruleus; LDTg, laterodorsal tegmental nucleus; LH, lateral hypothalamic area; LPAG, lateral periaqueductal gray; MPB, medial parabrachial nucleus; PCRt, parvicellular reticular nucleus; PH, posterior hypothalamic area; PnC, pontine reticular nucleus, caudal part; PnO, pontine reticular nucleus, oral part; PPTg, pedunculopontine tegmental nucleus; Pr, prepositus nucleus; RC, raphe cap; Sp5I spinal trigeminal nucleus, interpolar part; VLPAG, ventrolateral periaqueductal gray.

Serial coronal brain sections were imaged and brain structures were manually recognized by the atlas of mouse brain (36). We discovered that DsRed-labeled neurons were primarily located in the cerebellum and medulla. Only a few DsRed-labeled neurons were observed in the pons, midbrain, hypothalamus, thalamus, and cerebral cortex. Notably, DsRed-labeled neurons were primarily observed in the ipsilateral brain regions (Figure 3). To provide a detailed review of the presynaptic inputs, representative images were selected and enlarged, such as deep mesencephalic nucleus (DpMe), ventrolateral periaqueductal gray (VLPAG), parvicellular reticular nucleus (PCRt), dorsal raphe nucleus (DR), intermediate reticular nucleus (IRt), gigantocellular reticular nucleus (Gi), prepositus nucleus (Pr), locus coeruleus (LC), and dorsal paragigantocellular nucleus (DPGi) (Figure 4).

### 3.3 Commissural connections of GABAergic neurons in the MVN

**Figure 5:**
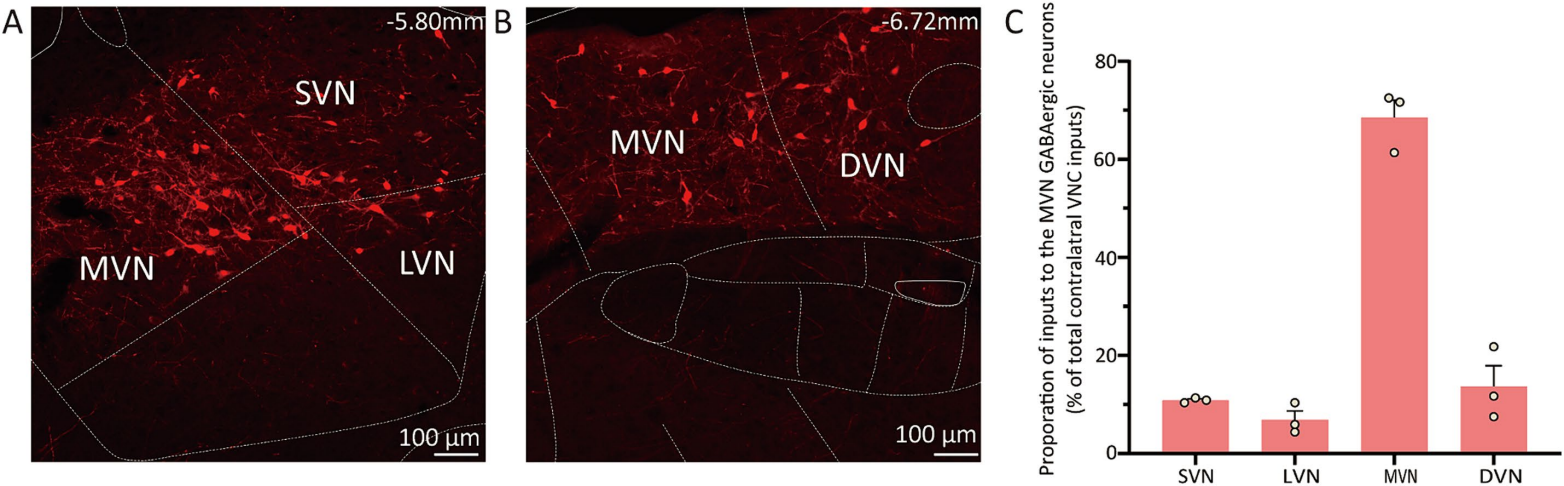
Connectivity between MVN GABAergic neurons and the contralateral VNC. **(A) (B)** Images showing dsRed-labeled neurons in contralateral MVN, LVN, SVN and DVN; **(C)** Statistical analysis of commissure connection (*n* = 3). VNC, vestibular nuclei complex; MVN, medial vestibular nucleus; LVN, lateral vestibular nucleus; SVN, superior vestibular nucleus; DVN, descending vestibular nucleus.

The contralateral vestibular nuclei complex (VNC), which includes the MVN, superior vestibular nucleus (SVN), lateral vestibular nuclei (LVN) and descending vestibular nucleus (DVN) was observed to reveal the commissural connection (37). The proportion of inputs from subnucleus was calculated as the count of DsRed-labeled cells in each subnucleus divided by the total count of DsRed-labeled cells in VNC. The MVN GABAergic neurons received most inputs from the contralateral MVN (68.54% ± 3.58%), as well as the contralateral DVN (13.68% ± 4.23%), SVN (10.87% ± 0.28%) and LVN (6.90% ± 1.79%) (Figure 5).

### 3.4 Analysis of afferent neurons providing input to MVN GABAergic neurons

We calculated the radio for each nucleus by dividing the count of DsRed-labeled neurons in a region by the total count of DsRed-labeled neurons ipsilaterally. We identified 60 nuclei projecting to MVN GABAergic neurons, each contributing over 0.1% of the total labeled neurons on the ipsilateral side. And proportions above 3% were defined as dense inputs, between 1 and 3% were defined as moderate inputs, and below 1% were defined as sparse inputs (35).

**Figure 6:**
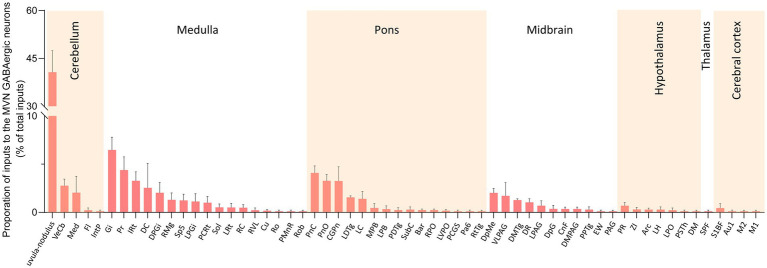
Statistical analysis of ipsilateral monosynaptic inputs to MVN GABAergic neurons. The average proportion of monosynaptic inputs from brain regions contributing more than 0.1% of the total inputs to MVN GABAergic neurons was analyzed and listed. Brain regions are categorized into seven general structures and presented at the top, Sample size: *n* = 3. Arc, arcuate hypothalamic nucleus; Bar, Barrington’s nucleus; Au1, primary auditory cortex; CGPn, central gray of the pons; CnF, cuneiform nucleus; Cu, cuneate nucleus; DC, dorsal cochlear nucleus; DM, dorsomedial hypothalamic nucleus; DMPAG, dorsomedial periaqueductal gray; DMTg, dorsomedial tegmental area; DpG, deep gray layer of the superior colliculus; DPGi, dorsal paragigantocellular nucleus; DpMe, deep mesencephalic nucleus; DR, dorsal raphe nucleus; EW, Edinger-Westphal nucleus; Fl, flocculus; Gi, gigantocellular reticular nucleus; IntP, interposed cerebellar nucleus, posterior part; IRt, intermediate reticular nucleus; LC, locus coeruleus; LDTg, laterodorsal tegmental nucleus; LH, lateral hypothalamic area; LPAG, lateral periaqueductal gray; MPB, medial parabrachial nucleus; LPGi, lateral paragigantocellular nucleus; LPO, lateral preoptic area; LRt, lateral reticular nucleus; LVPO, lateroventral periolivary nucleus; M1, primary motor cortex; M2, secondary motor cortex; Med, medial (fastigial) cerebellar nucleus; MPB, medial parabrachial nucleus; Pa6, paraabducens nucleus; PAG, periaqueductal gray; PCGS, paracochlear glial substance; PCRt, parvicellular reticular nucleus; PDTg posterodorsal tegmental nucleus; PMnR, paramedian raphe nucleus; PnC, pontine reticular nucleus, caudal part; PnO, pontine reticular nucleus, oral part; PPTg, pedunculopontine tegmental nucleus; Pr, prepositus nucleus; PR, prerubral field; PSTh, parasubthalamic nucleus; RC, raphe cap; RMg, raphe magnus nucleus; Ro, nucleus of Roller; Rob, raphe obscurus nucleus; RPO, rostral periolivary region; RtTg, reticulotegmental nucleus of the pons; RVL, rostroventrolateral reticular nucleus; S1BF, primary somatosensory cortex, barrel field; Sol, nucleus of the solitary tract; Sp5, spinal trigeminal tract; SPF, subparafascicular thalamic nucleus; SubC, subcoeruleus nucleus; VeCb, vestibulocerebellar nucleus; VLPAG, ventrolateral periaqueductal gray; ZI, zona incerta.

**Figure 7:**
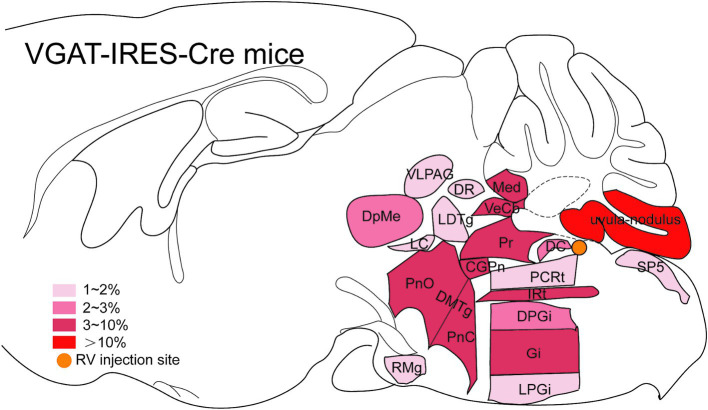
Schematic illustration showing the distribution of monosynaptic inputs to MVN GABAergic neurons. This figure provides a schematic illustration of the distribution patterns of monosynaptic inputs to MVN GABAergic neurons. The color density represents the amount of input neurons. CGPn, central gray of the pons; DC, dorsal cochlear nucleus; DMTg, dorsomedial tegmental area; DPGi, dorsal paragigantocellular nucleus; DpMe, deep mesencephalic nucleus; DR, dorsal raphe nucleus; Gi, gigantocellular reticular nucleus; IRt, intermediate reticular nucleus; LC, locus coeruleus; LDTg, laterodorsal tegmental nucleus; LPGi, lateral paragigantocellular nucleus; Med, medial (fastigial) cerebellar nucleus; PCRt, parvicellular reticular nucleus; PnC, pontine reticular nucleus, caudal part; PnO, pontine reticular nucleus, oral part; Pr, prepositus nucleus; RMg, raphe magnus nucleus; Sp5, spinal trigeminal tract; VeCb, vestibulocerebellar nucleus; VLPAG, ventrolateral periaqueductal gray.

Dense inputs (>3% of total DsRed-labeled neurons) to MVN GABAergic neurons originated from following nucleus: uvula-nodulus (40.675 ± 6.76%), Gi (6.48% ± 1.31%), Pr, (4.39 ± 1.27%), IRt (3.28% ± 0.93%), pontine reticular nucleus, caudal part (4.10% ± 0.73%), pontine reticular nucleus, oral part (3.28 ± 0.66%), central gray of the pons (3.25% ± 1.49%). Besides, MVN GABAergic neurons also received moderate monosynaptic inputs (more than 1% of total DsRed-labeled neurons) from several nuclei, such as: vestibulocerebellar nucleus, medial (fastigial) cerebellar nucleus (Med), dorsal cochlear nucleus, DPGi, raphe magnus nucleus, spinal trigeminal nucleus, lateral paragigantocellular nucleus (LPGi), PCRt, laterodorsal tegmental nucleus (LDTg), LC, DpMe, VLPAG, DR, lateral periaqueductal gray (LPAG) (Figure 6). A schematic diagram displaying the monosynaptic inputs to the MVN GABAergic neurons is shown in Figure 7.

## 4 Discussion

To gain a deeper insight of how MVN GABAergic neurons mediate physiological behaviors, it is necessary to explore the monosynaptic inputs to them which modulate their activity. In this study, a modified RV-based tracing system and VGAT-Cre mice were utilized. Our results revealed the presynaptic inputs to MVN GABAergic neurons, providing insight into the mechanisms mediating their activity. Additionally, we explored the commissural system and found that MVN GABAergic neurons are influenced by inputs from the contralateral MVN, LVN, SVN and DVN. These findings contribute to understanding commissure system and providing strategies to facilitate vestibular compensation.

### 4.1 Comparison with earlier tracing studies

Previous research in rats has revealed connectivity between the MVN and DR by using both the anterograde transport of biotinylated dextran amine and retrograde transport of Fluoro-Gold (27). This pathway was also confirmed on mice in our study. The traditional retrograde method using horseradish peroxidase showed the inferior olive (IO) projects to the MVN in rabbits (28, 30). However, we did not find specific inputs from the IO to the MVN GABAergic neurons, suggesting the IO may project to other neuron types of the MVN. This highlighted a limitation of traditional tracer methods which cannot identify cell type-specific neurons in the target nucleus. Genetically modified RV has been extensively used in anatomical studies, particularly in neurosciences, due to its effectiveness in labeling presynaptic inputs of defined neuronal cell-types in transgenic mice (38, 39).

To address this limitation, our group previously used the RV retrograde tracing system to investigate the monosynaptic inputs to GABAergic neurons in the VNC (32). However, heterogeneous subnuclei which performed distinct functions and commissure connections which attributed to vestibular compensation were not considered. In this study, we focused on the MVN, the largest subnucleus of the VNC. The RV-based retrograde system combined with VGAT-IRES-Cre mice was utilized to investigate the presynaptic inputs to GABAergic neurons of the MVN in this study. We discovered 60 upstream nuclei that innervated MVN GABAergic neurons, as well as inputs from the contralateral VNC that formed the commissural system. In conclusion, our study offered a more detailed and systematic mapping of inputs to MVN GABAergic neurons.

### 4.2 Implications for MVN GABAergic neurons in physiological behavior

The MVN neurons bilaterally travel through the medial longitudinal fasciculus to the medial ventral horn of the cervical cord. These neurons control the contraction of neck muscles to adjust the head and neck movements to maintain balance forming the vestibulospinal reflex. The MVN send ascending fibers to the ipsilateral oculomotor nucleus (CN 3) and contralateral abducens nucleus (CN 6) along with the SVN mediating the vestibuloocular reflex. This coordinate horizontal eye movements (40, 41).

Increased evidences have shown that the neurons connecting the MVN and the oculomotor nucleus was GABAergic, and these GABAergic neurons were also regulated by brain regions associated with oculomotor control (42, 43).

Results of this study confirmed this finding. The cerebellum gains direct projections from the vestibular end-organs and project to the MVN, acting as an adaptive processor (44–46). Direct inputs from the flocculus (Fl) and uvula-nodulus to the MVN have been revealed in cats and rabbits (1, 47–50). The cerebellum regulated the MVN through inhibitory inputs. Different regions projecting to MVN GABAergic neurons played distinct roles in regulating VOR. The unipolar brush cells within the uvula-nodulus receive vestibular inputs via mossy fibers from the vestibular end-organs and the vestibular nuclei. As feedback, these cells mediate the activity of the mossy fibers to control the vestibular inputs (51–55). Previous studies have shown damage of the uvula-nodulus affected the speed of the slow phase of eye movements relative to the head position, rather than the spatial orientation of the nystagmus (56, 57). Unlike the uvula-nodulus, the flocculus participated in the gain of the VOR (58). The Pr integrated the velocity and position signals of horizontal eye movements to maintain stable gaze (59). Researches in monkeys and humans have revealed the lesions of Pr results in defects in maintaining stable gaze (60–62). These indicated the uvula-nodulus, flocculus, and Pr are crucial components of the VOR circuits.

Additionally, sleep–wake system and vestibular system also interact. Clinically, patients with vestibular dysfunction often exhibit sleep disturbances, however, activation by electricity or rocking movements of the vestibular system can facilitate non-rapid eye movement (NREM) sleep (63–66). Franken and his colleagues found NREM sleep was increased and wakefulness episodes were shortened through stimulating the vestibular system by rocking movements at 1.0 Hz (67). Further studies revealed that neurotensinergic neurons in the MVN promoted NREM sleep, and these neurons were primarily GABAergic (68). By contrast, Yanagisawa et al. found that GABAergic neurons in the lateral MVN contributed to stabilizing wakefulness and regulating the transition into rapid eye movement (REM) sleep based on vestibular information (42). This may be reasonable because MVN GABAergic neurons were linked to various brain regions involved in not only improving sleep but also developing wakefulness. Likewise, MVN GABAergic neurons received direct projections from brain area related to sleep/wake cycle control. The LC and DR have been demonstrated to facilitate arousal (69, 70). Previous experiments showed there are projections from LC and DR to the vestibular nuclei (71, 72). In the present study, we further revealed the LC and DR send moderate projections to MVN GABAergic neurons. Inputs from the LC and DR can influence the gain of the vestibular reflexes and cerebellar-vestibular pathway, respectively (27, 73–75). In addition, afferent inputs to MVN GABAergic neurons also arose from NREM sleep-developing brain structures, such as the VLPAG and DpMe. The excitation of VLPAG GABAergic neurons increased NREM sleep and decreased REM sleep (76, 77). Chen et al. revealed that exciting GABAergic neurons in the dorsal part of DpMe promoted NREM sleep via the sublaterodorsal nucleus pathway (78). Brain nuclei that enhance REM sleep, such as the DPGi, LPGi, and LDTG, were found to send moderate inputs to the MVN GABAergic neurons in this study. DPGi GABAergic neurons enhanced REM sleep through the suppression of the LC and DR (79–81). Similarly, LPGi may hyperpolarize REM-off neurons in the LC to generate REM sleep (82). Electrical stimulation of LDTG also increased REM sleep (83).

GABAergic neurons in the MVN also participate in the vestibulosympathetic reflex to moderate blood distribution during postural change and movement. The MVN GABAergic neurons projected primarily to the caudal ventrolateral medulla (CVLM) which influenced sympathetic nerve activity by influencing the rostral ventrolateral medulla (23, 84). MVN GABAergic neurons receive feedback signals from sympathetic-related brain structures, such as the Gi and IRt. Kuo et al. found that activation of certain regions of the Gi induced a decrease in heart rate and caused hypotension in cats (85). The IRt served as a hub transmitting post-inspiratory activity to sympathetic and motor outputs (86, 87).

Our results revealed that MVN GABAergic neurons integrated multisensory signals related to oculomotor control, sleep/wakefulness regulation, and sympathetic responses. These findings established a basis for deeper investigation into the neural pathways mediating the physiological functions of MVN GABA neurons.

### 4.3 Implications for MVN GABAergic neurons in vestibular compensation

Normal vestibular system is essential for visual stabilization, postural maintenance, and equilibrium control, by relying on symmetrical afferent inputs to the vestibular nuclei (88). Several researches have shown that there are inter-nuclear connections between the bilateral vestibular nuclei (20, 89, 90). The inhibitory commissural system linking the MVN and its contralateral counterpart is fundamental to complete vestibular reflexes (91). Partial or total interruption of unilateral inputs, such as unilateral vestibular deafferentation (UVD) and unilateral labyrinthectomy led to postural and oculomotor deficits (92–94). These deficits were induced by the imbalance in activity between bilateral MVNs (95). The resting discharges of neurons in the ipsilesional MVN were almost silenced, whereas the contralesional MVN neurons became hyperactive (92, 96, 97). Another study demonstrated the resting potential of MVN neurons only decreased by 50% compared to normal situation after bilateral labyrinthectomy (98). These findings indicated that the silence of ipsilesional MVN neurons was primarily caused by enhanced suppression from contralesional MVN neurons (92). The vestibular dysfunction was characterized by static (without movement) and dynamic symptoms (with movement) (96). Static symptoms gradually disappeared within days known as vestibular compensation (91, 96, 99). Inhibitory commissural connections were crucial for the recovery of spontaneous resting potential of the lesioned side and rebalancing neural discharge between the bilateral MVN during vestibular compensation (91, 96).

Our findings showed that GABAergic neurons in the MVN were heavily innervated by projections from the contralateral MVN as well as the contralateral LVN, SVN, and DVN. These patterns were similar to the connections observed in hamsters, in contrast to the commissural connections in cats and monkeys showing afferents to the MVN arising from all parts of contralateral MVN, parts of contralateral SVN and DVN (71, 100). These discrepancies may be due to the differences between species. The commissural system to MVN GABAergic neurons revealed in the present study suggests that these neurons may be regulated by contralateral VNC to achieve bilateral balance, which was crucial for normal vestibular function.

In conclusion, we illustrated monosynaptic inputs to MVN GABAergic neurons. It suggested that MVN GABAergic neurons received information from various brain regions. This finding underscores the crucial role of MVN GABAergic neurons in integrating multiple signals. In addition, the confirmation of the commissure system provides provided evidences that MVN GABAergic neurons were involved in facilitating vestibular compensation.

## Data Availability

The raw data supporting the conclusions of this article will be provided by corresponding author without reservation.

## References

[1] Barmack NH. Central vestibular system: vestibular nuclei and posterior cerebellum. Brain Res Bull. (2003) 60:511–41. doi: 10.1016/S0361-9230(03)00055-8, PMID: 12787870

[2] Maklad A Fritzsch B. The developmental segregation of posterior crista and saccular vestibular fibers in mice: a carbocyanine tracer study using confocal microscopy. Brain Res Dev Brain Res. (2002) 135:1–17. doi: 10.1016/S0165-3806(01)00327-3, PMID: 11978388

[3] Cullen KE Zobeiri OA. Proprioception and the predictive sensing of active self-motion. Curr Opin Physio. (2021) 20:29–38. doi: 10.1016/j.cophys.2020.12.001, PMID: 33954270 PMC8095676

[4] Mildren RL Cullen KE. Vestibular contributions to primate neck postural muscle activity during natural motion. J Neurosci. (2023) 43:2326–37. doi: 10.1523/JNEUROSCI.1831-22.2023, PMID: 36801822 PMC10072293

[5] Cullen KE Wang L. Predictive coding in early vestibular pathways: implications for vestibular cognition. Cogn Neuropsychol. (2020) 37:423–6. doi: 10.1080/02643294.2020.1783222, PMID: 32619395 PMC7704804

[6] Horn AKE Straka H. Functional Organization of Extraocular Motoneurons and eye Muscles. Annu Rev Vis Sci. (2021) 7:793–825. doi: 10.1146/annurev-vision-100119-125043, PMID: 34524874

[7] Hitier M Besnard S Smith PF. Vestibular pathways involved in cognition. Front Integr Neurosci. (2014) 8:59. doi: 10.3389/fnint.2014.00059 25100954 PMC4107830

[8] Casani AP Gufoni M Capobianco S. Current insights into treating Vertigo in older adults. Drugs Aging. (2021) 38:655–70. doi: 10.1007/s40266-021-00877-z, PMID: 34159566 PMC8342368

[9] Tighilet B Chabbert C. Adult neurogenesis promotes balance recovery after vestibular loss. Prog Neurobiol. (2019) 174:28–35. doi: 10.1016/j.pneurobio.2019.01.001, PMID: 30658127

[10] Lacour M Tighilet B. Plastic events in the vestibular nuclei during vestibular compensation: the brain orchestration of a "deafferentation" code. Restor Neurol Neurosci. (2010) 28:19–35. doi: 10.3233/RNN-2010-0509, PMID: 20086280

[11] Lacour M Helmchen C Vidal PP. Vestibular compensation: the neuro-otologist's best friend. J Neurol. (2016) 263:54–S64. doi: 10.1007/s00415-015-7903-4 PMC4833803 27083885

[12] Diaz C Glover JC. The vestibular column in the mouse: a Rhombomeric perspective. Front Neuroanat. (2022) 15:806815. doi: 10.3389/fnana.2021.806815, PMID: 35173589 PMC8842660

[13] Tanaka I Ezure K. Overall distribution of GLYT2 mRNA-containing versus GAD67 mRNA-containing neurons and colocalization of both mRNAs in midbrain, pons, and cerebellum in rats. Neurosci Res. (2004) 49:165–78. doi: 10.1016/j.neures.2004.02.007, PMID: 15140559

[14] Walberg F Ottersen OP Rinvik E. GABA, glycine, aspartate, glutamate and taurine in the vestibular nuclei: an immunocytochemical investigation in the cat. Exp Brain Res. (1990) 79:547–63. doi: 10.1007/BF00229324, PMID: 1971225

[15] Kumoi K Saito N Tanaka C. Immunohistochemical localization of gamma-aminobutyric acid-and aspartate-containing neurons in the guinea pig vestibular nuclei. Brain Res. (1987) 416:22–33. doi: 10.1016/0006-8993(87)91492-2, PMID: 3304535

[16] Rácz E Gaál B Kecskes S Matesz C. Molecular composition of extracellular matrix in the vestibular nuclei of the rat. Brain Struct Funct. (2014) 219:1385–403. doi: 10.1007/s00429-013-0575-x, PMID: 23681169

[17] Gliddon CM Darlington CL Smith PF. GABAergic systems in the vestibular nucleus and their contribution to vestibular compensation. Prog Neurobiol. (2005) 75:53–81. doi: 10.1016/j.pneurobio.2004.11.001, PMID: 15713530

[18] Takazawa T Saito Y Tsuzuki K Ozawa S. Membrane and firing properties of glutamatergic and GABAergic neurons in the rat medial vestibular nucleus. J Neurophysiol. (2004) 92:3106–20. doi: 10.1152/jn.00494.2004, PMID: 15240763

[19] Saito Y Takazawa T Ozawa S. Relationship between afterhyperpolarization profiles and the regularity of spontaneous firings in rat medial vestibular nucleus neurons. Eur J Neurosci. (2008) 28:288–98. doi: 10.1111/j.1460-9568.2008.06338.x, PMID: 18702700

[20] Babalian A Vibert N Assie G Serafin M Mühlethaler M Vidal PP. Central vestibular networks in the guinea-pig: functional characterization in the isolated whole brain in vitro. Neuroscience. (1997) 81:405–26. doi: 10.1016/S0306-4522(97)00069-9, PMID: 9300431

[21] Blessing WW Hedger SC Oertel WH. Vestibulospinal pathway in rabbit includes GABA-synthesizing neurons. Neurosci Lett. (1987) 80:158–62. doi: 10.1016/0304-3940(87)90646-X, PMID: 3317134

[22] Precht W Baker R Okada Y. Evidence for GABA as the synaptic transmitter of the inhibitory vestibulo-ocular pathway. Exp Brain Res. (1973) 18:415–28. doi: 10.1007/BF00239109, PMID: 4360381

[23] Holstein GR Friedrich VL Jr Martinelli GP. Glutamate and GABA in Vestibulo-sympathetic pathway neurons. Front Neuroanat. (2016) 10:7. doi: 10.3389/fnana.2016.00007 26903817 PMC4744852

[24] Malinvaud D Vassias I Reichenberger I Rössert C Straka H. Functional organization of vestibular commissural connections in frog. J Neurosci. (2010) 30:3310–25. doi: 10.1523/JNEUROSCI.5318-09.2010, PMID: 20203191 PMC6634120

[25] Precht W Schwindt PC Baker R. Removal of vestibular commissural inhibition by antagonists of GABA and glycine. Brain Res. (1973) 62:222–6. doi: 10.1016/0006-8993(73)90631-8, PMID: 4765112

[26] Furuya N Yabe T Koizumi T. Neurotransmitters regulating vestibular commissural inhibition in the cat. Acta Otolaryngol Suppl. (1991) 481:205–8. doi: 10.3109/00016489109131381, PMID: 1681672

[27] Halberstadt AL Balaban CD. Organization of projections from the raphe nuclei to the vestibular nuclei in rats. Neuroscience. (2003) 120:573–94. doi: 10.1016/S0306-4522(02)00952-1, PMID: 12890525

[28] Balaban CD. Distribution of inferior olivary projections to the vestibular nuclei of albino rabbits. Neuroscience. (1988) 24:119–34. doi: 10.1016/0306-4522(88)90317-X, PMID: 3368043

[29] Balaban CD. Olivo-vestibular and cerebello-vestibular connections in albino rabbits. Neuroscience. (1984) 12:129–49. doi: 10.1016/0306-4522(84)90143-X, PMID: 6087195

[30] Balaban CD Kawaguchi Y Watanabe E. Evidence of a collateralized climbing fiber projection from the inferior olive to the flocculus and vestibular nuclei in rabbits. Neurosci Lett. (1981) 22:23–9. doi: 10.1016/0304-3940(81)90279-2, PMID: 7219886

[31] Balaban CD. Projections from the parabrachial nucleus to the vestibular nuclei: potential substrates for autonomic and limbic influences on vestibular responses. Brain Res. (2004) 996:126–37. doi: 10.1016/j.brainres.2003.10.026, PMID: 14670639

[32] Shi XB Wang J Li FT Zhang YB Qu WM Dai CF. Whole-brain monosynaptic outputs and presynaptic inputs of GABAergic neurons in the vestibular nuclei complex of mice. Front Neurosci. (2022) 16:982596. doi: 10.3389/fnins.2022.982596, PMID: 36090271 PMC9459096

[33] Ma WX Li L Kong LX Zhang H Yuan PC Huang ZL. Whole-brain monosynaptic inputs to lateral periaqueductal gray glutamatergic neurons in mice. CNS Neurosci Ther. (2023) 29:4147–59. doi: 10.1111/cns.14338, PMID: 37424163 PMC10651995

[34] Wickersham IR Lyon DC Barnard RJ Mori T Finke S Conzelmann KK. Monosynaptic restriction of transsynaptic tracing from single, genetically targeted neurons. Neuron. (2007) 53:639–47. doi: 10.1016/j.neuron.2007.01.033, PMID: 17329205 PMC2629495

[35] Shi X Wei H Chen Z Wang J Qu W Huang Z. Whole-brain monosynaptic inputs and outputs of glutamatergic neurons of the vestibular nuclei complex in mice. Hear Res. (2021) 401:108159. doi: 10.1016/j.heares.2020.108159, PMID: 33401198

[36] Paxinos G Franklin KBJ. The mouse brain in stereotaxic coordinates. 2nd ed. San Diego: Academic Press (2001).

[37] Straka H Dieringer N. Basic organization principles of the VOR: lessons from frogs. Prog Neurobiol. (2004) 73:259–309. doi: 10.1016/j.pneurobio.2004.05.003, PMID: 15261395

[38] Watabe-Uchida M Zhu L Ogawa SK Vamanrao A Uchida N. Whole-brain mapping of direct inputs to midbrain dopamine neurons. Neuron. (2012) 74:858–73. doi: 10.1016/j.neuron.2012.03.017, PMID: 22681690

[39] Pollak Dorocic I Fürth D Xuan Y Johansson Y Pozzi L Silberberg G. A whole-brain atlas of inputs to serotonergic neurons of the dorsal and median raphe nuclei. Neuron. (2014) 83:663–78. doi: 10.1016/j.neuron.2014.07.002, PMID: 25102561

[40] Khan S Chang R. Anatomy of the vestibular system: a review. Neuro Rehabil. (2013) 32:437–43. doi: 10.3233/NRE-130866, PMID: 23648598

[41] McCall AA Miller DM Yates BJ. Descending influences on Vestibulospinal and Vestibulosympathetic reflexes. Front Neurol. (2017) 8:112. doi: 10.3389/fneur.2017.00112 28396651 PMC5366978

[42] Nakatsuka D Kanda T Sato M Ishikawa Y Cherasse Y Yanagisawa M. A novel GABAergic population in the medial vestibular nucleus maintains wakefulness and gates rapid eye movement sleep. iScience. (2024) 27:109289. doi: 10.1016/j.isci.2024.109289, PMID: 38482494 PMC10933495

[43] Wentzel PR De Zeeuw CI Holstege JC Gerrits NM. Inhibitory synaptic inputs to the oculomotor nucleus from vestibulo-ocular-reflex-related nuclei in the rabbit. Neuroscience. (1995) 65:161–74. doi: 10.1016/0306-4522(94)00471-G, PMID: 7538643

[44] Cullen KE. Internal models of self-motion: neural computations by the vestibular cerebellum. Trends Neurosci. (2023) 46:986–1002. doi: 10.1016/j.tins.2023.08.009, PMID: 37739815 PMC10591839

[45] Zobeiri OA Cullen KE. Distinct representations of body and head motion are dynamically encoded by Purkinje cell populations in the macaque cerebellum. eLife. (2022) 11:e75018. doi: 10.7554/eLife.75018, PMID: 35467528 PMC9075952

[46] Zobeiri OA Cullen KE. Cerebellar Purkinje cells in male macaques combine sensory and motor information to predict the sensory consequences of active self-motion. Nat Commun. (2024) 15:4003. doi: 10.1038/s41467-024-48376-0, PMID: 38734715 PMC11088633

[47] Barmack NH Qian Z Yoshimura J. Regional and cellular distribution of protein kinase C in rat cerebellar Purkinje cells. J Comp Neurol. (2000) 427:235–54. doi: 10.1002/1096-9861(20001113)427:2<235::AID-CNE6>3.0.CO;2-6, PMID: 11054691

[48] Shojaku H Sato Y Ikarashi K Kawasaki T. Topographical distribution of Purkinje cells in the uvula and the nodulus projecting to the vestibular nuclei in cats. Brain Res. (1987) 416:100–12. doi: 10.1016/0006-8993(87)91501-0, PMID: 3620947

[49] Walberg F Dietrichs E. The interconnection between the vestibular nuclei and the nodulus: a study of reciprocity. Brain Res. (1988) 449:47–53. doi: 10.1016/0006-8993(88)91022-0, PMID: 2456133

[50] Fujita H Kodama T du Lac S. Modular output circuits of the fastigial nucleus for diverse motor and nonmotor functions of the cerebellar vermis. eLife. (2020) 9:e58613. doi: 10.7554/eLife.58613, PMID: 32639229 PMC7438114

[51] Elliott KL Kersigo J Lee JH Yamoah EN Fritzsch B. Sustained loss of Bdnf affects peripheral but not central vestibular targets. Front Neurol. (2021) 12:768456. doi: 10.3389/fneur.2021.768456, PMID: 34975728 PMC8716794

[52] Pan N Jahan I Lee JE Fritzsch B. Defects in the cerebella of conditional Neurod 1 null mice correlate with effective Tg (Atoh 1-cre) recombination and granule cell requirements for Neurod 1 for differentiation. Cell Tissue Res. (2009) 337:407–28. doi: 10.1007/s00441-009-0826-6, PMID: 19609565 PMC3023111

[53] Maklad A Fritzsch B. Partial segregation of posterior crista and saccular fibers to the nodulus and uvula of the cerebellum in mice, and its development. Brain Res Dev Brain Res. (2003) 140:223–36. doi: 10.1016/S0165-3806(02)00609-0, PMID: 12586428

[54] Balmer TS Trussell LO. Selective targeting of unipolar brush cell subtypes by cerebellar mossy fibers. eLife. (2019) 8:e44964. doi: 10.7554/eLife.44964, PMID: 30994458 PMC6469928

[55] Diño MR Perachio AA Mugnaini E. Cerebellar unipolar brush cells are targets of primary vestibular afferents: an experimental study in the gerbil. Exp Brain Res. (2001) 140:162–70. doi: 10.1007/s002210100790, PMID: 11521148

[56] Errico P Ferraresi AA Barmack NH Pettorossi VE. Role of cerebellar uvula-nodulus in the control of head orientation-specific eye velocity in the rabbit. Ann N Y Acad Sci. (1996) 781:614–8. doi: 10.1111/j.1749-6632.1996.tb15738.x, PMID: 8694455

[57] Voogd J Barmack NH. Oculomotor cerebellum. Prog Brain Res. (2006) 151:231–68. doi: 10.1016/S0079-6123(05)51008-2 16221591

[58] Chang HHV Cook AA Watt AJ Cullen KE. Loss of Flocculus Purkinje cell firing precision leads to impaired gaze stabilization in a mouse model of spinocerebellar Ataxia type 6 (SCA6). Cells. (2022) 11:2739. doi: 10.3390/cells11172739, PMID: 36078147 PMC9454745

[59] Kim SH Zee DS du Lac S Kim HJ Kim JS. Nucleus prepositus hypoglossi lesions produce a unique ocular motor syndrome. Neurology. (2016) 87:2026–33. doi: 10.1212/WNL.0000000000003316, PMID: 27733568 PMC5109954

[60] Kaneko CR. Eye movement deficits after ibotenic acid lesions of the nucleus prepositus hypoglossi in monkeys. I Saccades and fixation. J Neurophysiol. (1997) 78:1753–68. doi: 10.1152/jn.1997.78.4.1753, PMID: 9325345

[61] Kaneko CR. Eye movement deficits following ibotenic acid lesions of the nucleus prepositus hypoglossi in monkeys II. Pursuit, vestibular, and optokinetic responses. J Neurophysiol. (1999) 81:668–81. doi: 10.1152/jn.1999.81.2.668, PMID: 10036269

[62] Cho HJ Choi HY Kim YD Seo SW Heo JH. The clinical syndrome and etiological mechanism of infarction involving the nucleus prepositus hypoglossi. Cerebrovasc Dis. (2008) 26:178–83. doi: 10.1159/000145325, PMID: 18628616

[63] Kim SK Kim JH Jeon SS Hong SM. Relationship between sleep quality and dizziness. PLoS One. (2018) 13:e0192705. doi: 10.1371/journal.pone.0192705, PMID: 29513688 PMC5841657

[64] Nakayama M Suzuki M Inagaki A Takemura K Watanabe N Tanigawa T. Impaired quality of sleep in Ménière's disease patients. J Clin Sleep Med. (2010) 6:445–9. doi: 10.5664/jcsm.27933 20957844 PMC2952747

[65] van Sluijs RM Rondei QJ Schluep D Jäger L Riener R Achermann P. Effect of rocking movements on afternoon sleep. Front Neurosci. (2020) 13:1446. doi: 10.3389/fnins.2019.01446, PMID: 32038144 PMC6985453

[66] Goothy SSK McKeown J. Modulation of sleep using electrical vestibular nerve stimulation prior to sleep onset: a pilot study. J Basic Clin Physiol Pharmacol. (2020) 32:19–23. doi: 10.1515/jbcpp-2020-0019, PMID: 33006952

[67] Kompotis K Hubbard J Emmenegger Y Perrault A Mühlethaler M Schwartz S. Rocking promotes sleep in mice through rhythmic stimulation of the vestibular system. Curr Biol. (2019) 29:392–401.e4. doi: 10.1016/j.cub.2018.12.007, PMID: 30686738

[68] Kashiwagi M Kanuka M Tatsuzawa C Suzuki H Morita M Tanaka K. Widely distributed Neurotensinergic neurons in the brainstem regulate NREM sleep in mice. Curr Biol. (2020) 30:1002–1010.e4. doi: 10.1016/j.cub.2020.01.047, PMID: 32032507

[69] Carter ME Yizhar O Chikahisa S Nguyen H Adamantidis A Nishino S. Tuning arousal with optogenetic modulation of locus coeruleus neurons. Nat Neurosci. (2010) 13:1526–33. doi: 10.1038/nn.2682, PMID: 21037585 PMC3174240

[70] Ito H Yanase M Yamashita A Kitabatake C Hamada A Suhara Y. Analysis of sleep disorders under pain using an optogenetic tool: possible involvement of the activation of dorsal raphe nucleus-serotonergic neurons. Mol Brain. (2013) 6:59. doi: 10.1186/1756-6606-6-59, PMID: 24370235 PMC3879646

[71] Horowitz SS Blanchard J Morin LP. Medial vestibular connections with the hypocretin (orexin) system. J Comp Neurol. (2005) 487:127–46. doi: 10.1002/cne.20521, PMID: 15880498

[72] Schuerger RJ Balaban CD. Immunohistochemical demonstration of regionally selective projections from locus coeruleus to the vestibular nuclei in rats. Exp Brain Res. (1993) 92:351–9. PMID: 8095905 10.1007/BF00229022

[73] Schuerger RJ Balaban CD. Organization of the coeruleo-vestibular pathway in rats, rabbits, and monkeys. Brain Res Brain Res Rev. (1999) 30:189–217. doi: 10.1016/S0165-0173(99)00015-6, PMID: 10525175

[74] Foote SL Bloom FE Aston-Jones G. Nucleus locus ceruleus: new evidence of anatomical and physiological specificity. Physiol Rev. (1983) 63:844–914. doi: 10.1152/physrev.1983.63.3.844, PMID: 6308694

[75] Kishimoto T Sasa M Takaori S. Inhibition of lateral vestibular nucleus neurons by 5-hydroxytryptamine derived from the dorsal raphe nucleus. Brain Res. (1991) 553:229–37. doi: 10.1016/0006-8993(91)90830-O, PMID: 1933282

[76] Weber F Hoang Do JP Chung S Beier KT Bikov M Doost MS. Regulation of REM and non-REM sleep by periaqueductal GABAergic neurons. Nat Commun. (2018) 9:354. doi: 10.1038/s41467-017-02765-w, PMID: 29367602 PMC5783937

[77] Weber F Chung S Beier KT Xu M Luo L Dan Y. Control of REM sleep by ventral medulla GABAergic neurons. Nature. (2015) 526:435–8. doi: 10.1038/nature14979, PMID: 26444238 PMC4852286

[78] Chen ZK Dong H Liu CW Liu WY Zhao YN Xu W. A cluster of mesopontine GABAergic neurons suppresses REM sleep and curbs cataplexy. Cell Discov. (2022) 8:115. doi: 10.1038/s41421-022-00456-5, PMID: 36280664 PMC9592589

[79] Fraigne JJ Torontali ZA Snow MB Peever JH. REM sleep at its Core-circuits, neurotransmitters, and pathophysiology. Front Neurol. (2015) 6:123. doi: 10.3389/fneur.2015.00123 26074874 PMC4448509

[80] Gervasoni D Peyron C Rampon C Barbagli B Chouvet G Urbain N. Role and origin of the GABAergic innervation of dorsal raphe serotonergic neurons. J Neurosci. (2000) 20:4217–25. doi: 10.1523/JNEUROSCI.20-11-04217.2000, PMID: 10818157 PMC6772634

[81] Ennis M Aston-Jones G. GABA-mediated inhibition of locus coeruleus from the dorsomedial rostral medulla. J Neurosci. (1989) 9:2973–81. doi: 10.1523/JNEUROSCI.09-08-02973.1989, PMID: 2769374 PMC6569704

[82] Sirieix C Gervasoni D Luppi PH Léger L. Role of the lateral paragigantocellular nucleus in the network of paradoxical (REM) sleep: an electrophysiological and anatomical study in the rat. PLoS One. (2012) 7:e28724. doi: 10.1371/journal.pone.0028724, PMID: 22235249 PMC3250413

[83] Thakkar M Portas C McCarley RW. Chronic low-amplitude electrical stimulation of the laterodorsal tegmental nucleus of freely moving cats increases REM sleep. Brain Res. (1996) 723:223–7. doi: 10.1016/0006-8993(96)00256-9, PMID: 8813404

[84] Holstein GR Friedrich VL Jr Martinelli GP. Projection neurons of the vestibulo-sympathetic reflex pathway. J Comp Neurol. (2014) 522:2053–74. doi: 10.1002/cne.23517, PMID: 24323841 PMC3997612

[85] Kuo JS Hwa Y Chai CY. Cardio-inhibitory mechanism in the gigantocellular reticular nucleus of the medulla oblongata. Brain Res. (1979) 178:221–32. doi: 10.1016/0006-8993(79)90691-7, PMID: 509206

[86] Toor RUA Sun QJ Kumar NN Le S Hildreth CM Phillips JK. Neurons in the intermediate reticular nucleus coordinate Postinspiratory activity, swallowing, and respiratory-sympathetic coupling in the rat. J Neurosci. (2021) 41:1617. doi: 10.1523/JNEUROSCI.0502-19.2019 31666354 PMC6891060

[87] Huff A Karlen-Amarante M Oliveira LM Ramirez JM. Role of the postinspiratory complex in regulating swallow-breathing coordination and other laryngeal behaviors. eLife. (2023) 12:e86103. doi: 10.7554/eLife.86103, PMID: 37272425 PMC10264072

[88] Swamy Suman N Kumar Rajasekaran A Yuvaraj P Pruthi N Thennarasu K. Measure of central vestibular compensation: a review. J Int Adv Otol. (2022) 18:441–6. doi: 10.5152/iao.2022.21207, PMID: 35971266 PMC9524397

[89] Newlands SD Kevetter GA Perachio AA. A quantitative study of the vestibular commissures in the gerbil. Brain Res. (1989) 487:152–7. doi: 10.1016/0006-8993(89)90951-7, PMID: 2752282

[90] Epema AH Gerrits NM Voogd J. Commissural and intrinsic connections of the vestibular nuclei in the rabbit: a retrograde labeling study. Exp Brain Res. (1988) 71:129–46. doi: 10.1007/BF00247528, PMID: 2458274

[91] Graham BP Dutia MB. Cellular basis of vestibular compensation: analysis and modelling of the role of the commissural inhibitory system. Exp Brain Res. (2001) 137:387–96. doi: 10.1007/s002210100677, PMID: 11355384

[92] Paterson JM Menzies JRW Bergquist F Dutia MB. Cellular mechanisms of vestibular compensation. Neuroembryol Aging. (2006) 3:183–93. doi: 10.1159/000096796

[93] Curthoys IS. Vestibular compensation and substitution. Curr Opin Neurol. (2000) 13:27–30. doi: 10.1097/00019052-200002000-00006 10719646

[94] Dieringer N. Activity-related postlesional vestibular reorganization. Ann N Y Acad Sci. (2003) 1004:50–60. doi: 10.1196/annals.1303.006 PMID: 14662447

[95] Fisch U. The vestibular response following unilateral vestibular neurectomy. Acta Otolaryngol. (1973) 76:229–38. doi: 10.3109/00016487309121503 4542914

[96] Jones SM Jones TA Mills KN Gaines GC. Anatomical and physiological considerations in vestibular dysfunction and compensation. Semin Hear. (2009) 30:231–41. doi: 10.1055/s-0029-1241124, PMID: 21072129 PMC2975108

[97] Smith PF Curthoys IS. Mechanisms of recovery following unilateral labyrinthectomy: a review. Brain Res Brain Res Rev. (1989) 14:155–80. doi: 10.1016/0165-0173(89)90013-1, PMID: 2665890

[98] Ris L Godaux E. Neuronal activity in the vestibular nuclei after contralateral or bilateral labyrinthectomy in the alert guinea pig. J Neurophysiol. (1998) 80:2352–67. doi: 10.1152/jn.1998.80.5.2352 9819248

[99] Tighilet B Lacour M. Gamma amino butyric acid (GABA) immunoreactivity in the vestibular nuclei of normal and unilateral vestibular neurectomized cats. Eur J Neurosci. (2001) 13:2255–67. doi: 10.1046/j.0953-816x.2001.01622.x, PMID: 11454029

[100] Carleton SC Carpenter MB. Afferent and efferent connections of the medial, inferior and lateral vestibular nuclei in the cat and monkey. Brain Res. (1983) 278:29–51. doi: 10.1016/0006-8993(83)90223-8, PMID: 6315158

